# High incidence of silent venous thromboembolism before treatment in ovarian cancer

**DOI:** 10.1038/sj.bjc.6603989

**Published:** 2007-09-25

**Authors:** T Satoh, A Oki, K Uno, M Sakurai, H Ochi, S Okada, R Minami, K Matsumoto, Y O Tanaka, H Tsunoda, S Homma, H Yoshikawa

**Affiliations:** 1 Department of Obstetrics and Gynecology, Institute of Clinical Medicine, Graduate School of Comprehensive Human Sciences, University of Tsukuba, Ibaraki, Japan; 2 Cardiovascular Division, Institute of Clinical Medicine, Graduate School of Comprehensive Human Sciences, University of Tsukuba, Ibaraki, Japan; 3 Department of Radiology, Institute of Clinical Medicine, Graduate School of Comprehensive Human Sciences, University of Tsukuba, Ibaraki, Japan; 4 Department of Obstetrics and Gynecology, Kanto Medical Center NTT EC, Tokyo, Japan

**Keywords:** ovarian cancer, deep-vein thrombosis, pulmonary thromboembolism, plasma D-dimer, clear cell adenocarcinoma

## Abstract

Venous thromboembolism (VTE) such as deep-vein thrombosis (DVT) and pulmonary thromboembolism (PTE) often occurs after surgery and rarely occurs even before surgery in patients with ovarian cancer. It is well known that levels of plasma D-dimer (DD) before treatment in most ovarian cancer patients are increased. This study therefore examined whether increased levels of DD are associated with presence of VTE before treatment of ovarian cancer. Between November 2004 and March 2007, DD levels prior to initial treatment were measured in 72 consecutive patients with presumed epithelial ovarian cancer (final diagnosis: epithelial ovarian cancer, *n*=60; and epithelial ovarian borderline malignancy, *n*=12). Venous ultrasound imaging (VUI) of the lower extremity was conducted for all patients except for two patients in whom DVT was detected by pelvic computed tomography (CT). When DVT was found, pulmonary scintigraphy was subsequently performed to ascertain presence of PTE. D-dimer levels were above the cut-off value (0.5 *μ*g ml^−1^) in 65 of 72 patients (90.2%). Venous ultrasound imaging or CT revealed DVT in 18 of 72 patients (25.0%) and pulmonary scintigraphy found PTE in 8 patients (11.1%). All patients with VTE were asymptomatic when VTE was found. D-dimer levels were associated with incidence of VTE (0–1.4 *μ*g ml^−1^; 0 of 26 (0%), 1.5–7.4 *μ*g ml^−1^; 9 of 30 (30%) and ⩾7.5 *μ*g ml^−1^; 9 of 16 (56.3%), *P* for trend=0.0003). However, even if 1.5 *μ*g ml^−1^ was used as a cut-off value, this had low specificity and positive predictive value (47.2, 38.3%), though it had high sensitivity and negative predictive value (100, 100%). Therefore, ovarian cancer patients with DD level ⩾1.5 *μ*g ml^−1^ should be examined using VUI to detect silent DVT. Patients with VTE underwent preventive managements including anticoagulant therapy before initial treatment, chemotherapy or surgery, and after surgery. There was no clinical onset of postoperative VTE in all 72 patients. Measurement of DD levels and subsequent ultrasonography revealed that silent or subclinical VTE frequently occurs before surgery in ovarian cancer. The usefulness of preoperative assessment of VTE needs further confirmation in randomised controlled trials.

Deep-vein thrombosis (DVT) and subsequent pulmonary thromboembolism (PTE) represent potentially lethal perioperative complications associated with surgery for ovarian cancer (von Tempelhoff *et al*, 1998). Deep-vein thrombosis is known to occur in the postoperative period or during the course of postoperative chemotherapy in 13.6–27.0% of ovarian cancer patients (Clarke-Pearson *et al*, 1983; von Tempelhoff *et al*, 1998). Several guidelines (Greets *et al*, 2001; Nicolaides *et al*, 2001) have focused on intra- and postoperative managements for prevention of venous thromboembolism (VTE). However, large tumours or massive ascites in ovarian cancer may compress the intrapelvic veins and increase the risk of DVT even before surgery. For prevention of DVT after surgery, we usually use elastic stockings during surgery and intermittent pneumatic compression (IPC) during and after surgery. However, if DVT exists before treatment of ovarian cancer, such preventative measures may be ineffective or possibly dangerous for lethal PTE.

D-dimer (DD) is a degradation product of fibrin and reflects fibrin concentration. Several studies have reported that levels of plasma DD before treatment in most ovarian cancer patients are increased and related to advanced disease, suggesting DD as a useful tumour marker or prognostic factor of ovarian cancer (Mirshahi *et al*, 1992; Gadducci *et al*, 1996). Although high levels of DD are generally thought to be associated with the presence of DVT (Bounameaux *et al*, 1991; Harrison *et al*, 1993; Wells *et al*, 2003; Righini *et al*, 2006), one study reported that preoperatively increased DD levels in ovarian cancer are not significantly associated with risk of subsequent DVT in the postoperative period and during first-line chemotherapy (von Tempelhoff *et al*, 1997). However, patients in that study received either low molecular weight heparin or unfractionated heparin as perioperative anticoagulant therapy, starting 2 h before surgery and continued until postoperative day 7. That study did not examine associations between increased DD levels and presence of silent VTE before surgery.

We undertook the present study to clarify whether increased levels of DD are associated with presence of silent VTE before treatment for ovarian cancer and whether pretreatment assessment of silent VTE can contribute to the prevention of clinical onset of VTE after surgery for ovarian cancer.

## MATERIALS AND METHODS

### Patients

The study protocol was approved by the Ethical Committee of the University of Tsukuba Hospital. Between November 2004 and March 2007, a total of 72 consecutive patients with presumed epithelial ovarian cancer were enrolled in this study. All patients provided written informed consent. Initial treatment was performed in the Department of Obstetrics and Gynecology at the University of Tsukuba Hospital. D-dimer levels were measured on the first visits to our outpatient clinic 2–5 weeks before initial treatment of ovarian cancer. Tumour of 60 patients were histologically confirmed to be ovarian cancer after surgery. The remaining 12 patients underwent primary surgery and tumours were histologically diagnosed as ovarian borderline malignancy (serous type, *n*=2; mucinous type, *n*=9; endometrioid type, *n*=1). Histology of the ovarian cancers was as follows: serous adenocarcinoma, *n*=31; mucinous adenocarcinoma, *n*=6; endometrioid adenocarcinoma, *n*=5; clear cell adenocarcinoma, *n*=14; and undifferentiated carcinoma, *n*=4. Criteria of the International Federation of Gynecology and Obstetrics (FIGO; 1988) were used to classify the 60 patients with ovarian cancer as stage I (*n*=13), stage II (*n*=13), stage III (*n*=24) or stage IV (*n*=10), while the 12 patients with ovarian borderline malignancy all had stage I disease. Mean age of patients was 54.5 years (range: 17–87 years). Mean body mass index (BMI) of patients was 23.2 (range: 13.7–35.0). Mean tumour size before treatment was 14.0 cm (range: 1.4–43.0 cm) according to maximum diameter as evaluated on magnetic resonance imaging (MRI).

### Measurement of plasma DD level

Peripheral blood samples were collected from all patients before treatment, and DD levels were measured. Blood samples were drawn from an antecubital vein with atraumatic puncture into plastic tubes using a two-tube technique, discarding the first 4–5 ml. Whole blood was anticoagulated by addition of 9 volumes to 1 volume of 0.11 mol l^−1^ sodium citrate solution, then centrifuged at 3000 r.p.m. for 10 min. Citrate plasma was then removed and frozen at −20°C for up to 3 days before assessment.

D-dimer levels were measured using STA-Liatest D-Di latex (Diagnostica Stago, Asnières, France) sensitised with anti-DD mouse monoclonal antibody to induce a latex coagulation reaction, and turbidity was quantified using a spectrophotometer. The cut-off value of DD level in this method is 0.5 *μ*g ml^−1^.

### Detection of DVT

All patients underwent comprehensive imaging of abdominal extension of the tumour and presence of thrombus in iliac veins and inferior vena cava using computed tomography (CT) and MRI. Venous ultrasound imaging (VUI) was performed to detect DVT in all patients except for two patients who were found to have DVT in common and external iliac veins on pelvic-enhanced CT. Ultrasonography was performed using an ATL HDI5000 system (Philips Medical Systems, Bothell, WA, USA) equipped with a 3- to 7.5-MHz transducer. Power, pulse repetition frequency and wall thump filter settings were adjusted for venous vascular studies. Iliac, femoral, great saphenous, popliteal, peroneal, posttibial and soleal veins were evaluated bilaterally. Iliac and femoral veins were assessed in a supine position and other veins were assessed in an upright position. All veins were imaged on transverse and long-axis views. Venous lumens were observed while searching for thrombus by manual compression with transducer and colour Doppler signals. For evaluation of intrapelvic veins, reactions during Valsalva manoeuvre were also observed. No reaction during Valsalva manoeuvre was considered to represent suspected proximal venous flow disturbance, and we diagnosed intrapelvic DVT based on the results of enhanced CT.

### Pulmonary perfusion scintigraphy

In principle, patients with DVT detected by pelvic CT or VUI were examined by pulmonary perfusion scintigraphy using Tc-99 to evaluate the presence of PTE. However, four patients who displayed old and organised thrombus in popliteal or soleal veins were omitted from scintigraphy due to the low risk for PTE.

### Management of VTE and ovarian cancer

We performed anticoagulant therapy using unfractionated heparin before initial treatment and after surgery for all patients with VTE. In addition, one of the following managements was selected in some patients: placement of inferior vena cava filter (IVCF) before upfront surgery or neoadjuvant chemotherapy followed by interval debulking surgery.

Inferior vena cava filter placement was used for preventing lethal PTE in patients, with DVT in proximal veins such as iliac and femoral veins or floating DVT in peripheral veins, who underwent upfront surgery.

Neoadjuvant chemotherapy followed by interval debulking surgery was selected for patients with apparent stage III/IV ovarian cancer and chemosensitive tumour histology, that is, serous or endometrioid type as estimated on CT, excessively increased CA125 level and cytological findings of ascites. We administered TC regimen consisting of paclitaxel (175 mg m^−2^ and over 3-h infusion) and AUC 6 of carboplatin as neoadjuvant chemotherapy.

We also used TC regimen as postoperative chemotherapy in patients with stages Ic–IV ovarian cancer. Patients with ovarian borderline malignancy did not undergo chemotherapy.

## RESULTS

### Patient characteristics and risk of VTE

Table 1 shows the relationship between patient characteristics and risk of VTE (DVT and/or PTE) before treatment. Deep-vein thrombosis was found in 18 of 72 patients (25.0%) and PTE was detected in 8 of these 18 patients (DVT alone, *n*=10; DVT plus PTE, *n*=8). Though patients with DVT included two patients with ovarian borderline malignancy, the two patients did not have PTE. Study variables included age, FIGO stage, histology, BMI, presence of massive ascites and tumour size. Among these variables, clear cell histology was significantly associated with both DVT and PTE, and presence of massive ascites was significantly associated with DVT. Patients with clear cell histology displayed a higher incidence of VTE compared to those with nonclear histology (DVT, 50.0 *vs* 19.0%; PTE, 35.7 *vs* 5.2%; Table 1). The multivariate analysis for massive ascites and clear cell histology confirmed that both factors were independently and significantly associated with the risk of DVT (*P*=0.013 and 0.006).

### Plasma level of DD

**Figure 1:**
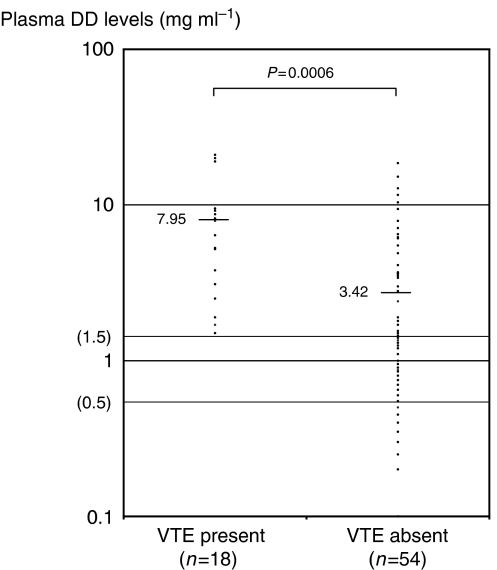
Plasma D-dimer levels before treatment of ovarian cancer and borderline malignancy in patients with and without VTE.

D-dimer levels were above the reference value (0.5 *μ*g ml^−1^) in 65 of out 72 patients with ovarian cancer and borderline malignancy (90.3%). Figure 1 shows DD levels in patients with and without VTE (DVT or DVT plus PE). D-dimer levels were significantly higher in patients with VTE than in patients without VTE (mean and range of plasma DD levels, 7.95 *μ*g ml^−1^ (1.5–20.0 *μ*g ml^−1^) *vs* 3.42 *μ*g ml^−1^ (0.2–18.6 *μ*g ml^−1^); *P*=0.01). If 1.5 *μ*g ml^−1^ was used as a cut-off value for DD levels to diagnose VTE, sensitivity, specificity and positive and negative predictive values were 100, 47.2, 38.3 and 100% (Table 2). D-dimer levels were associated with incidence of VTE (0–1.4 *μ*g ml^−1^; 0 out of 26 (0%), 1.5–7.4 *μ*g ml^−1^; 9 out of 30 (30%) and ⩾7.5 *μ*g ml^−1^; 9 out of 16 (56.3%), *P* for trend=0.0003). Increased DD levels were associated with higher incidence of VTE (Table 3).

### DVT and PE

Deep-vein thrombosis was found in 16 patients with ovarian cancer and 2 patients with ovarian borderline malignancy. Pulmonary thromboembolism was confirmed in eight patients with ovarian cancer. All patients with DVT alone and DVT plus PTE were asymptomatic when DVT or PTE was found. However, only one patient with DVT and PTE experienced dyspnoea the day after pulmonary scintigraphy and was admitted to our hospital on emergency.

The most proximal location of DVT was the common iliac vein in one patient, external iliac vein in one patient, femoral vein in one patient, saphenous vein in one patient, peroneal vein in seven patients, posttibial vein in one patient and soleal vein in six patients. For two patients with ovarian borderline malignancy, one had DVT in the saphenous vein and one had DVT in the soleal vein.

### Management of VTE and cancer treatment

Anticoagulant therapy was performed before initial treatment and after upfront or interval debulking surgery for all 18 patients with VTE. In addition, we used placement of IVCF or neoadjuvant chemotherapy in eight of these patients according to severity of VTE, stage of ovarian cancer, expected histology of the tumour, cytological evidence of malignancy and performance status of patients. As a result, we managed patients with VTE as follows: (1) anticoagulant therapy alone in 10 patients including 2 patients with ovarian borderline malignancy; (2) upfront debulking surgery with placement of IVCF in 4 patients; and (3) neoadjuvant chemotherapy followed by IDS in 4 patients.

As a result of these mentioned managements, none of these patients developed clinical manifestations of VTE after surgery.

## DISCUSSION

The present study frequently detected silent VTE before treatment of ovarian cancer and borderline malignancy. Several studies have documented preoperative DVT and PTE in patients with ovarian cancer (Goff *et al*, 1996; Mitsui *et al*, 2001; Jain *et al*, 2002). One study reported that preoperative plasma DD level was high in two patients who developed PTE postoperatively. High incidence of silent VTE before surgery seems attributable to the high incidence of VTE after surgery in ovarian cancer. Our data suggest that silent or subclinical VTE frequently occurs before surgery, and progresses and clinically manifests after surgery in ovarian cancer.

Among six variables considered as risk factors for VTE, clear cell histology and presence of massive ascites were significantly associated with VTE before treatment of ovarian cancer. Several previous reports have postulated that patients with clear cell adenocarcinoma experience a significantly higher incidence of VTE (11–42%) compared to those with nonclear carcinoma in the immediate postoperative period and during primary chemotherapy (Goff *et al*, 1996; Recio *et al*, 1996). In the present study, clear cell histology was a risk factor for both preoperative silent DVT and PTE in ovarian cancer patients. Very recently, we used immunohistochemical methods to reveal that clear cell adenocarcinoma shows significantly stronger expression of tissue factor, a major factor in the procoagulant activities of cancer cells, compared to nonclear cell adenocarcinoma (Uno *et al*, 2007). Massive ascites was also an independent and significant risk factor for preoperative DVT in ovarian cancer. One explanation is that massive ascites may increase blood viscosity in blood vessels due to dehydration. Clear cell histology and massive ascites seem to represent independent and significant risk factors for silent VTE before treatment and probably also for VTE after surgery in ovarian cancer.

We revealed that increased levels of DD are associated with presence of silent VTE before treatment of ovarian cancer. A suitable cut-off value for detecting VTE in patients with ovarian cancer seems to be 1.5 *μ*g ml^−1^, as this had a 100% sensitivity and a 100% negative predictive value. However, this cut-off value had only a 47.2% specificity. Therefore, ovarian cancer patients with DD level ⩾1.5 *μ*g ml^−1^ should be examined using VUI of the lower extremity to detect silent DVT.

In the guidelines for preventing VTE following general surgery reported at the 6th American College of Chest Physicians Consensus Conference (Greets *et al*, 2001), patients with malignant ovarian tumour are classified as the highest risk group. These guidelines focused on prevention of postoperative VTE and recommended the use of elastic stockings and IPC during and after surgery and anticoagulant therapy after surgery in patients with ovarian cancer. The guidelines included no recommendations regarding assessment of silent VTE in patients. However, the present study revealed that patients tend to have VTE such as DVT and PTE even before treatment of ovarian cancer. There is a possibility that the management based on preoperative assessment of VTE may contribute to prevention of clinical manifestation of VTE after surgery.

In conclusion, measurement of DD level and subsequent ultrasonography revealed that silent or subclinical VTE frequently occurs before surgery in ovarian cancer. The usefulness of preoperative assessment of VTE needs further confirmation in randomised controlled trials.

## Figures and Tables

**Table 1:** Incidence of deep-vein thrombosis and pulmonary thromboembolism by patient characteristics with ovarian cancer and borderline tumour

**Characteristics**	**DVT**	**OR (95% CI)**	***P*-value**	**PTE**	**OR (95% CI)**	***P*-value**
*Age (years)*						
<50	5/23 (21.7%)	Reference		3/23 (13.0%)	Reference	
⩾50	13/49 (26.5%)	1.30 (0.40–4.21)	0.66	5/49 (10.2%)	0.76 (0.16–3.49)	0.66
						
*BMI*						
<25	14/53 (26.4%)	Reference		7/53 (13.2%)	Reference	
⩾25	4/19 (21.1%)	0.74 (0.21–2.62)	0.64	1/19 (5.3%)	0.36 (0.04–3.18)	0.34
						
*FIGO stage (1989)*						
Stage I/II	9/38 (23.7%)	Reference		5/38 (13.2%)	Reference	
Stage III/IV	9/34 (26.5%)	1.16 (0.40–3.37)	0.79	3/34 (8.8%)	0.63 (0.14–2.90)	0.56
						
*Histology*						
Serous carcinoma	5/31 (16.1%)	Reference		1/31 (3.2%)	Reference	
CCC	7/14 (50.0%)	5.20 (1.26–21.5)	0.02	5/14 (35.7%)	16.7 (1.72–161)	<0.01
Others	4/15 (26.7%)	1.89 (0.43–8.41)	0.40	2/15 (13.3%)	4.62 (0.38–55.5)	0.51
Borderline tumour	2/12 (16.7%)	1.04 (0.17–6.26)	0.97	0/12 (0.00%)	ND	
						
Non-CCC	11/58 (19.0%)	Reference		3/58 (5.2%)	Reference	
CCC	7/14 (50.0%)	4.27 (1.24–14.7)	0.02	5/14 (35.7%)	10.2 (2.07–50.2)	<0.01
						
*Massive ascites* a						
Absent	10/54 (18.5%)	Reference		6/54 (11.1%)	Reference	
Present	8/18 (44.4%)	3.52 (1.11–11.2)	0.03	2/18 (11.1%)	1.00 (0.18–5.46)	1.00
						
*Tumour size (cm)*						
<10	6/26 (23.1%)	Reference		3/26 (11.5%)	Reference	
⩾10	12/46 (26.1%)	1.18 (0.38–3.62)	0.78	5/46 (10.9%)	0.93 (0.20–4.27)	0.93

BMI=body mass index; CCC=clear cell carcinoma; CT=computed tomography; DVT=deep-vein thrombosis; FIGO=International Federation of Gynecology and Obstetrics; ND=not done; OR=odds ratio; 95% CI=95% confidence interval; PTE=pulmonary thromboembolism.

a Massive ascites was defined as centralisation detected by CT in this study.

**Table 2:** Sensitivity, specificity and positive and negative predictive values of different cut-off D-dimer levels for diagnosis of deep-vein thrombosis before treatment for ovarian cancer and borderline malignancy

**Cut-off D-dimer level (*μ*g ml^−1^)**	**Sensitivity (%)**	**Specificity (%)**	**PPV (%)**	**NPV (%)**
0.5	100	12.7	27.3	100
1.5	100	47.2	38.3	100
3.0	77.8	61.8	40.0	89.5
4.5	66.7	76.4	48.0	87.5
6.0	55.6	80.0	47.6	84.3
7.5	50.0	85.5	52.9	83.9

NPV=negative predictive value; PPV=positive predictive value.

**Table 3:** Incidence of deep-vein thrombosis for each level of D-dimer with ovarian cancer and borderline tumour (*P* for trend=0.0003)

	**Incidence of VTE**		
**DD level (*μ*g ml^−1^)**	**Ovarian cancer**	**Borderline malignancy**	**Total**
0.0–1.4	0/21 (0%)	0/5 (0%)	0/26 (0%)
1.5–7.4	7/23 (30.4%)	2/7(28.6%)	9/30 (30.0%)
⩾7.5	9/16 (56.3%)	0/0	9/16 (56.3%)

DD=D-dimer; VTE=venous thromboembolism.
